# Handwritten-Digit Recognition by Hybrid Convolutional Neural Network based on HfO_2_ Memristive Spiking-Neuron

**DOI:** 10.1038/s41598-018-30768-0

**Published:** 2018-08-22

**Authors:** J. J. Wang, S. G. Hu, X. T. Zhan, Q. Yu, Z. Liu, T. P. Chen, Y. Yin, Sumio Hosaka, Y. Liu

**Affiliations:** 1 State Key Laboratory of Electronic Thin Films and Integrated Devices, University of Electronic Science and Technology of China, Chengdu, 610054 P. R. China; 2 School of Materials and Energy, Guangdong University of Technology, Guangzhou, 510006 P. R. China; 3 School of Electrical and Electronic Engineering, Nanyang Technological University, Singapore, 639798 Singapore; 4 Graduate School of Engineering, Gunma University, 1-5-1Tenjin, Kiryu, Gunma, 376-8515 Japan

## Abstract

Although there is a huge progress in complementary-metal-oxide-semiconductor (CMOS) technology, construction of an artificial neural network using CMOS technology to realize the functionality comparable with that of human cerebral cortex containing 10^10^–10^11^ neurons is still of great challenge. Recently, phase change memristor neuron has been proposed to realize a human-brain level neural network operating at a high speed while consuming a small amount of power and having a high integration density. Although memristor neuron can be scaled down to nanometer, integration of 10^10^–10^11^ neurons still faces many problems in circuit complexity, chip area, power consumption, etc. In this work, we propose a CMOS compatible HfO_2_ memristor neuron that can be well integrated with silicon circuits. A hybrid Convolutional Neural Network (CNN) based on the HfO_2_ memristor neuron is proposed and constructed. In the hybrid CNN, one memristive neuron can behave as multiple physical neurons based on the Time Division Multiplexing Access (TDMA) technique. Handwritten digit recognition is demonstrated in the hybrid CNN with a memristive neuron acting as 784 physical neurons. This work paves the way towards substantially shrinking the amount of neurons required in hardware and realization of more complex or even human cerebral cortex level memristive neural networks.

## Introduction

The human brain, consisting of 10^10^–10^11^ neurons and 10^14^–10^15^ synapses, has powerful cognitive capability while consuming low power (~15 watts)^1^. Inspired by the advanced information processing scheme in the brain, neuromorphic hardware using artificial neurons and synapses has aroused extensive concern in recent years^2^. Although improvements in CMOS device integration have been achieved by periodic doubling transistor density over the past half century^3^, it is still challenging to implement a neuromorphic hardware based on the standard CMOS technology to achieve the functionality comparable to the human brain. For example, the TrueNorth neuromorphic chip with 5.4 × 10^9^ transistors can realize 1 × 10^6^ spiking neurons and 2.56 × 10^8^ synapses only^4^. In order to construct a brain-level neuromorphic hardware, it is inevitable to develop nanometer-scale neuromorphic devices and the Time Division Multiplexing Access (TDMA) technique may be a possible pathway for shrinking the amount of physical neurons.

Recently, great effort has been made to develop neuromorphic devices that can emulate the behaviors of neuronal systems. Among the emerging neuromorphic devices (e.g., neuron transistor^5^, phase-change device^6^, ferroelectric device^7^, magnetic device^8^), memristors whose resistance can be quasi-continuously adjusted by electrical stimuli, have attracted the most attention^9,10^. After Williams *et al*. announced the discovery of memristor in 2008^11–13^, it has been demonstrated that memristors can effectively emulate synaptic plasticity, such as the long-term potentiation/depression^14,15^ and spike-timing dependent plasticity^16,17^, as well as brain-like behaviors, such as learning and forgetting functions^18,19^. More recently, small scale neuromorphic hardware using memristors as synapses have been demonstrated to realize brain-like behaviors. M. Ziegler *et al*. and Y. V. Pershin *et al*. respectively reported a simple neural network consisting of three electronic neurons connected by memristor-based synapses for implementing associative learning like the Pavlov’s dog^20,21^. S. B. Eryilmaz *et al*. and S. G. Hu *et al*. respectively realized associative memory in Hopfield neural networks based on memristor synapses^22,23^. D. B. Strukov *et al*. realized 33-pixel pattern classification in memristive crossbar circuits via *ex situ* or *in situ* training^24–26^. A. Serb *et al*. demonstrated pattern classification through unsupervised learning in a winner-take-all neural network based on multi-state memristive synapses^27^. P. Yao implemented 3-class face classification in a 1024-cell one transistor and one memristor (1T1R) synapse array^28^. W. D. Lu *et al*. demonstrated image reconstruction in a 32 × 32 memristor crossbar hardware through sparse coding^29^.

In the year of 2016, researchers from IBM reported a stochastic neuron with neuronal state directly stored in a phase-change device^30^; subsequently they presented neuromorphic architectures comprising neurons and synapses based on phase-change devices in the tasks of correlation detection^31–33^, pattern learning and feature extraction^34^. Although the phase change spiking neuron shows good neuromorphic properties, the memristor using CMOS-compatible materials is more promising and competitive for constructing spiking neuron because of its simple structure, excellent scalability, low cost, and good compatibility with standard CMOS processes^35,36^. On the other hand, in order to implement memristive neural network, building important types of artificial neural networks based on memristive neurons is necessary. Convolutional neural network (CNN) is a variant of multilayer perceptron (MLP) inspired by the organization of the animal visual cortex^37^, which has been widely applied in many artificial intelligent fields, such as handwritten numeral recognition^38^, object detection^39^, video classification^40^, and etc. Demonstration of building CNN based on memristive neuron may pave the way for further extensive use of memristive neural network in artificial intelligence. Most recently, fully integrated memristive neural networks were realized, which provide a more efficient approach to implementing neural network algorithms than traditional hardware^41^. L. Ni *et al*. achieved high energy efficiency and throughput in a ReRAM-based binary CNN accelerator^42^ and proposed an area-saving matrix-vector multiplication accelerator based on binary RRAM crossbar, which exhibits superior performance and low power consumption on machine learning^43^.

In this work, a CMOS-compatible HfO_2_ memristor neuron, which has the ability to mimic the integration of pre-synapse current in membrane of biological neuron, is designed and implemented. A hybrid CNN based on the HfO_2_ memristor neuron has been constructed. In the network, one memristive neuron can behave as 784 physical neurons based on the Time Division Multiplexing Access (TDMA) technique. Handwritten digits can be recognized by using the CNN. This study provides the possibility of both a substantial reduction of the amount of physical neurons required in the hardware and realization of more complex or even human brain level memristive neural network.

## Materials and Methods

The memristor used as neuron is based on a metal-insulator-metal (MIM) structure with a thin HfO_2_ layer as the insulator. The device fabrication details were described in our previous work^23^. Dies with different electrode areas were cut from the wafer and packaged in standard 28-pin dual in-line package (DIP) for constructing the memristive neuron. The hybrid Convolutional Neural Network (CNN) based on the memristive neuron was realized on a printed circuit board (PCB) (see Supplementary Figure 1). The CNN consists of one memristive neuron, four transmission-gate chips (Maxim MAX313), seven operational amplifiers (Texas Instruments OPA4322AIPWR), and one comparator chip (Texas Instruments LM339). Electrical characteristics of the memristor were measured with a Keithley 4200 semiconductor characterization system at room temperature. In the measurement of the CNN, “Raspberry PI” PAD minicomputer was used to generate the clock signals, and the waveforms of the clock signals and outputs were recorded with a RIGOL oscilloscope (model No. DS4024) and was analyzed by “Respberry PI” PAD minicomputer. Details of TDMA is provided in Supplementary Note 2.

## Results

**Figure 1:**
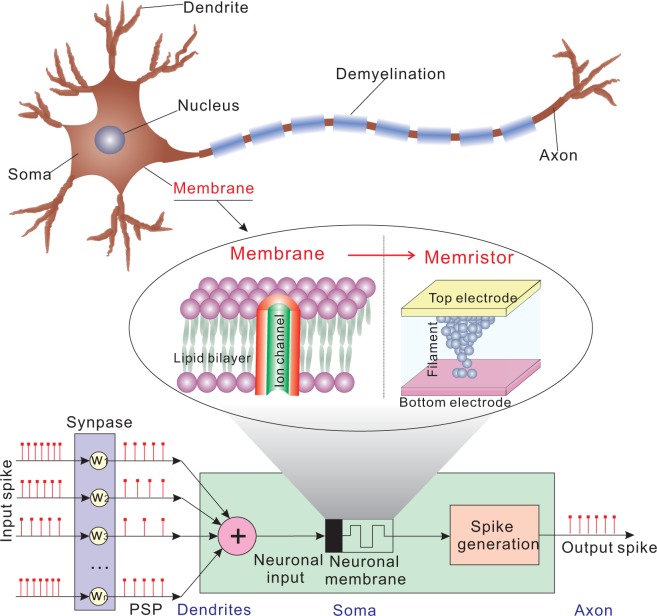
An artificial neuron based on a HfO_2_ memristor. Schematic of an artificial neuron that consists of the dendrites (inputs), the soma (which comprises the neuronal membrane and the spike event generation) and the axon (output). The dendrites may be connected to synapses in a network. The input spikes are modulated by synapse in frequency domain having the same amplitude.

In conventional artificial neuron, capacitors are normally used as the neural membrane. However, capacitors occupy a large area, resulting in impossibility to integrate a large amount of neurons in a chip. Memristors have much higher integration density and consume less energy. Besides, they are compatible with CMOS technology and have higher speed than capacitor-based neuron. In addition, although the phase-change memristive neuron shows good neuronal properties^30^, neuron based on the fully CMOS-compatible material HfO_2_ is still preferred considering the integration and process compatibility. HfO_2_ is a high-k material which has been widely used to replace SiO_2_ as the gate dielectric in CMOS technology (i.e., 28 nm CMOS technology^44^). And thus, the memristor based on HfO_2_ thin film is completely compatible with the standard CMOS technology. Besides, memristor based on HfO_2_ shows superior performances^45^, such as excellent radiation resistance, endurance and retention characteristics. Last but not least, the neuron based on the HfO_2_ exhibits similar properties of uncertainty analogous to biological neuron, which plays a key role in signal encoding and transmission^46^. The memristor having the hafnium oxide sandwiched between two metal electrodes is illustrated in Figure 1. The conductive filament model can be used to explain the change of resistance/conductance in the HfO_2_ memristor. Detailed device structure and conductive switching mechanism were described in our previous work^23^. As shown in Figure 1, an artificial spike-based neuron based on the HfO_2_ memristor containing dendrites (inputs), soma, and axon (output) has been designed and implemented in this work. The dendrite is connected to synapse for communication with other neurons. The soma comprises the neuronal membrane and the spike generation module. In a nonlinear integrate-and-fire neuron model, the membrane potential y can be expressed as

1 $$\frac{dy}{dt}=-\,L(y)+g(y,t)\cdot u$$

where *L*(*y*) is the leaky term, reflecting the imperfections in membrane; *g*(*y*, *t*) is the conductance of the ion channel representing the synaptic weight; and *u* denotes the voltage due to the arrival of the pre-synapse spike. The artificial neuron follows equation (1) until the membrane potential increases beyond a threshold. Then the soma fires a spike and resets the neuron to the initial state. More information about the memristive neuron circuit design and typical waveforms are provided in Supplementary Note 3.

**Figure 2:**
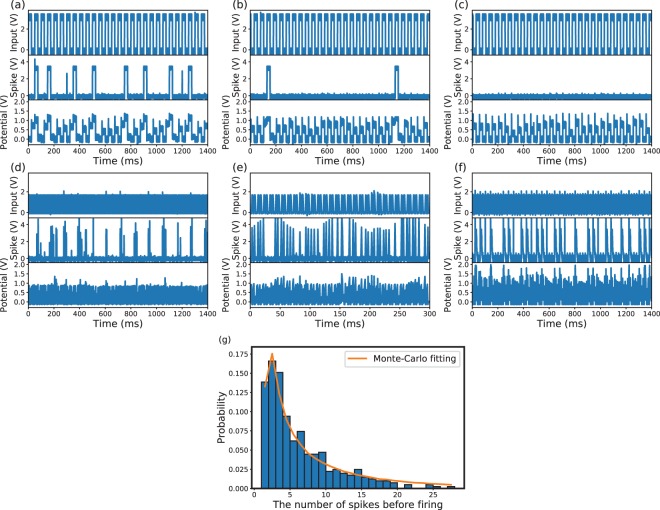
Characteristics of the spiking-neuron based on HfO_2_ memristor. Neuron response for input when V_th_ = 0.5 V (**a**), 0.8 V(**b**), and 1.0 V (**c**) at 100 Hz; neuron response for V_th_ = 0.7 V at 10 Hz (**d**), 100 Hz (**e**), and 1 kHz (**f**); and (**g**) firing possibility for the number of spikes.

The integration functionality of neuron can be realized by applying successive pulses to the memristor. The signals of dendrites (inputs), axon (output) and somas (membrane potential) are shown in Figure 2. The electrical potential of the memristor increases step by step until a dendrite spike occurs, which reflects the integration functionality in membrane. When there is no dendrite spike applying to the memristor, a fixed current is applied to the memristor for sensing the resistance of the memristor. After several pulses, the resistance of the memristor increases beyond the threshold analogous to the threshold potential in a biological neuron. Then an axon spike is fired by the neuron. At the same time, a pulse is applied to the memristor to reset the memristor to the low-resistance state.

Figure 2a, 2b and 2c show the neuron firing properties for different thresholds of 0.5 V, 0.8 V and 1.0 V, respectively. As can be observed, a higher threshold leads to less axon spikes occurrence. The input spiking frequency also takes effect on the integration in the memristive neuron. Figure 2d, 2e and 2f show the neuron firing properties for different input spiking frequencies of 10 Hz, 100 Hz and 1 kHz, respectively. Higher input frequency of spikes causes more output spikes. On the other hand, real biological neural activity may show significant irregularities due to intrinsic and extrinsic neural noise that originates from the biochemical processes of the individual neuron in network. The memristive neuron implemented in this work also exhibits similar properties of uncertainty analogous to biological neurons. In some circumstances, the resistance of the memristor is unchanged for most spikes; while it suddenly approaches the high-resistance state for some spikes. The randomness of neuron firing is shown in Figure 2g. The Monte-Carlo simulation is carried out to fit the experimental result of the memristive neuron firing. As can be seen in Figure 2g, the Monte-Carlo fitting result is close to the actual histogram obtained from the memristive neuron firing experiments. The random integration-and-fire behavior of memristive neuron is caused by the random resetting event of memristor instead of the real integration. Figure 2g indicates that the designed memristive neuron can emulate real biological neurons well in uncertainty. More information about neuron firing properties is presented in Supplementary Note 4.

**Figure 3:**
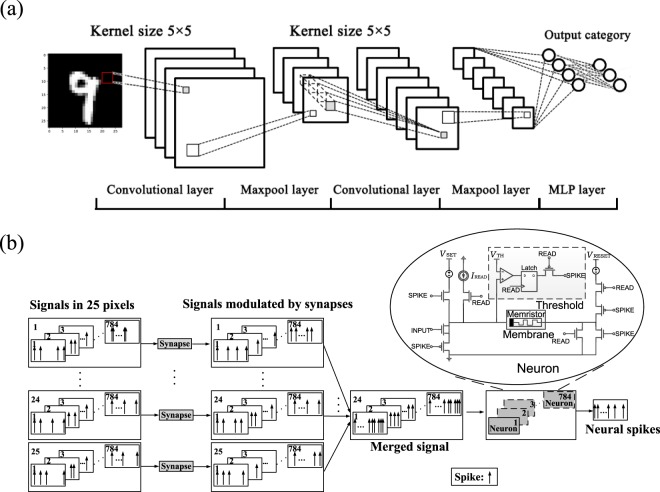
Convolutional neural network based on memristive neuron. (**a**) Illustration of the CNN when recognizing digit “9”; (**b**) illustration of one memristive neuron working as 784 neurons in the first convolutional layer in the CNN based on TDMA.

Using the memristive neuron, a hybrid convolutional neural network was constructed to demonstrate digit recognition. More CNN design information is provided in Supplementary Note 5. A “Raspberry PI” minicomputer was used to generate dendrite spikes and receive output axon spikes from neurons. In the hybrid neural network, neurons can either be the memristive neurons or software-based. The two types of neurons are exchangeable to verify the amount of neurons that the memristive neuron can behave as based on TDMA. It is should be noted that by using TDMA technique the speed of CNN is reduced as the network must spend time in accessing the same memristive neuron in series. Trade-off between the CNN speed and the number of the memristive neuron required should be considered. On the other hand, in the hybrid network synapses, maximum function and activation function are implemented in software domain by “Raspberry PI” minicomputer. The system photograph is presented in Supplementary Figure 1. As shown in Figure 3a, 5 × 5 synapses are connected to a neuron in the convolutional layer, and all of the neurons share the same synaptic weight in a certain layer due to the CNN inherent characteristics. Due to the shared synaptic weight, a single memristive neuron can behave as many neurons based on TDMA technique as shown in Figure 3b. The neural network was trained on Graphic Processing Unit (GPU) computing platform with Tensorflow^47^. After training, the convolutional kernels and weight matrixes are transferred into the hybrid CNN synaptic matrix. The information about training of the CNN is provided in Supplementary Note 6.

As shown in Figure 3a, the inference process is realized by the CNN with 6 layers including 2 convolutional layers, 2 maxpool layers and 2 fully connected layers. In the convolutional layers, the photographs of the handwritten digits are divided into several overlapping grids. In the first convolutional layer the photograph is divided into 28 × 28 grids and each grid contains 5 × 5 pixels. Zeros are filled at photograph borders to make the amount of grids equaled to that of the original photograph. The output of a grid is the weighted sum of the pixels; and the network inputs the sum to the activation function of the rectified linear unit (ReLU function). The outputs of all grids are made of a transformed photograph containing abstracted information. For the first convolutional layer, 32 convolutional kernels are used, and thus 32 different abstracted photographs (channels) of the handwritten digits are obtained. The convolutional layer is followed by a maxpool layer to reduce redundant information as well as the amount of pixels. The maxpool layer receives all pixels of one channel generated by the convolutional layer, and divides the pixels into several non-overlapping grids. In the first maxpool layer, the channel is divided into 14 × 14 grids; and each grid contains 2 × 2 pixels. The output of the first maxpool layer also contains 32 channels, each of which has 14 × 14 pixels. The second convolutional layer is the same as the first one except that the amount of convolutional kernels is 64. Before the fully connected layer, the output of maxpool layer is reshaped into one dimensional array, whose amount of elements is 3136 (7 × 7 × 64). As shown in Figure 3a, the convolutional layers are followed by a 2 layers of fully connected perceptron, which contains 1024 neurons and 10 neurons, respectively. Each neuron in perceptron receives the spikes, calculates the weighted sum of the spike and transfers the sum to the nonlinear activation function (ReLU function) to obtain the output. Handwritten digit recognition inference process is carried out according to:

2 $${f}_{out}(i,j)=\sum _{{k}_{i}=0}^{4}\,\sum _{{k}_{j}=0}^{4}[{w}_{{k}_{i}{k}_{j}}\cdot {f}_{in}(i+{k}_{i},j+{k}_{j}])]$$

3 $${f}_{out}(i,j)=\mathop{{\rm{\max }}}\limits_{2i\le {p}_{i}\le 2i+2}[\mathop{{\rm{\max }}}\limits_{2j\le {p}_{j}\le 2j+2}{f}_{in}({p}_{i},{p}_{j})]$$

4 $${f}_{out}(i,j)=\sum _{i=0}^{n}[{w}_{i}\cdot {f}_{in}(i)]$$

Equation (2) presents convolution operation in convolutional layer, where w_kikj_ is the convolutional kernel and *f*_*in*_(*i*, *j*) is the frequency of the input spike. Equation (3) presents the maxpool operation following the One-Hot rule (See Supplementary Note 5). The operation in fully connected layer follows equation (4), where *w*_*i*_ is the i^th^ weight of perceptron. Considering the characteristics of the memristive neuron, all signals received by neurons are modulated by frequency. The gray scale of handwritten photograph is modulated to a fixed amplitude spike, and the frequency of spike is proportional to the gray scale level. Considering the connection between synapses and neurons, the software-based synapse in the network is designed to be a frequency modulator. Synapses receive the spike sequence, and modify the frequency of the pulse sequence according to the pre-defined weight, and send out output spiking sequence. The calculation in the network follows equations (2–4). The detailed information of the output of channels in each layer when recognizing handwritten “9” is presented in Supplementary Note 7.

In the inference process, considering the time domain delay factor, the memristive neuron behaves as 784 neurons in first convolutional layer based on TDMA technique, as shown in Figure 3b. Neurons in other layers are software-based and they are replicable by the memristive neuron. The photographs of the handwritten of digits in the test set of MNIST database^48^ were used to examine the hybrid CNN. Ten thousand different photographs were examined through this CNN, and the accuracy rate is round 97.1% which approaches the rate of a pure software based network through GPU. More information about the CNN recognition of handwritten “9” is provided in Supplementary Note 8.

**Figure 4:**
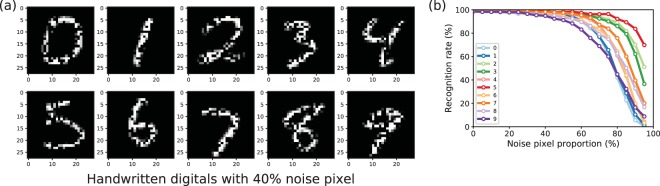
Handwritten digit recognition by the CNN based on memristive neuron. (**a**) an example of handwritten digits with 40% noise pixel enrolled; (**b**) the recognition rate for digits “0–9” as functions of noise pixel proportion.

Figure 4a shows examples of noisy photographs for the digits “0” to “9”. Noises are added by randomly choosing pixels and assigning them to zero. Figure 4b shows the accuracy rate on ten thousand of test images with a certain proportion of noise. The average accuracy rate is more than 90% for the image which is distorted by adding 40% noise. In this work, all weights in convolutional layers and fully connection layers are positive for easy application in hardware.

## Discussion

In summary, CMOS compatible neuron has been realized based on HfO_2_ memristor. We have demonstrated a hybrid CNN based on the HfO_2_ memristive neuron. One memristive neuron is used behaving as 784 neurons in the convolutional layer in the neural network based on the TDMA technique. Handwritten digits can be recognized through this memristive CNN. This study provides the possibility to reduce the number of neurons required by more than two orders as well as to realize hardware implementation of brain-level artificial neuromorphic networks.

## Electronic supplementary material

Supplementary Information
