# Supplementary material for: Handwritten-Digit Recognition by Hybrid Convolutional Neural Network based on HfO2 Memristive Spiking-Neuron
Source: Sci Rep. 2018 Aug 22;8:12546. doi: 10.1038/s41598-018-30768-0 (PMC6105732; doi:10.1038/s41598-018-30768-0)
Supplement: Supplementary file 1 — Supplementary Information [file 41598_2018_30768_MOESM1_ESM.docx]

Handwritten-Digit Recognition by Hybrid Convolutional Neural Network based on HfO_2_ Memristive Spiking-Neuron

J. J. Wang†^1^, S. G. Hu†^1^, X. T. Zhan^1^, Q. Yu^1^, Z. Liu^2^, T. P. Chen^3^, Y. Yin^4^ , Sumio Hosaka^4^ and Y. Liu^*1^

^1^State Key Laboratory of Electronic Thin Films and Integrated Devices, University of Electronic Science and Technology of China, Chengdu 610054, P. R. China

^2^School of Materials and Energy, Guangdong University of Technology, Guangzhou 510006, P. R. China

^3^School of Electrical and Electronic Engineering, Nanyang Technological University, Singapore 639798

^4^Graduate School of Engineering, Gunma University, 1-5-1Tenjin, Kiryu, Gunma 376-8515, Japan

†These authors contribute equally to this work

^*^Corresponding Author E-mail: yliu1975@uestc.edu.cn

**SUPPLEMENTARY INFORMATION**

**Supplimentary Note 1: Memristor device and hardwares**


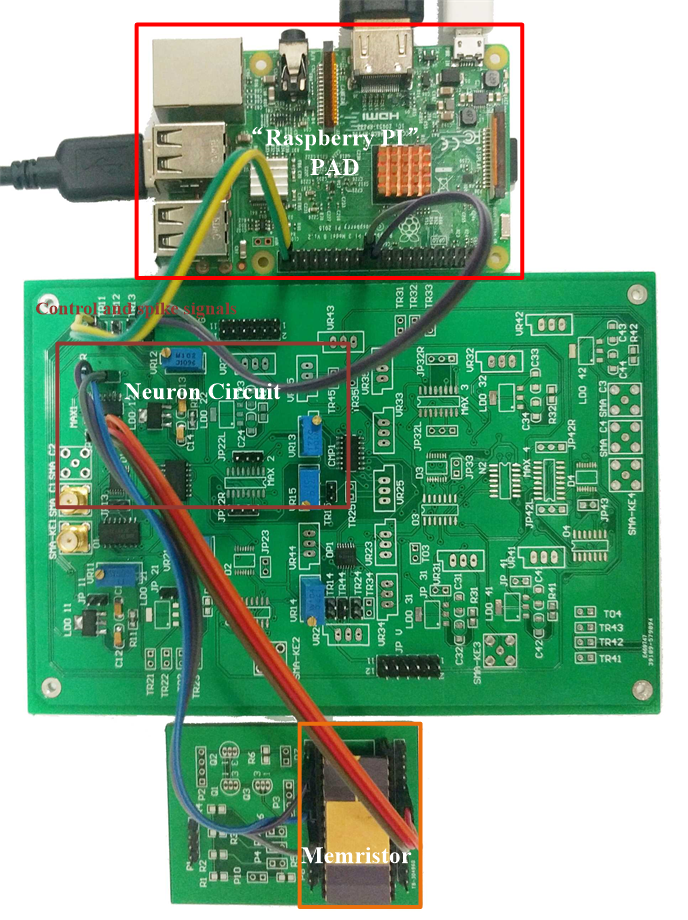


Supplimentary Figure 1. Photograph of the overall hybrid CNN based on the memristive neuron.

**Supplimentary Note 2: Circuit design of memristor neuron**

The circuit implementation of the memristor neuron is shown in Supplimentary Figure 2a. A hybrid circuit that contains analog switches and digital elements (logic gates and D-Flip-flops) is designed to implement the integrate-fire-and-reset functionality of neuron. In the circuit, the “read” signal is the opposite of input signal generated by an inverter. The proposed design takes advantage of no extra synchronous signal needed. After initialization, the neuron is ready to receive spikes from pre-synapse. The resistance of memristor can be modified only when the input is active. When no spike received, I_READ_ is applied to the memristor to estimate the memristor resistance. If the resistance of memristor increases beyond the threshold which leads to the anode voltage beyond V_th,_ the firing event is then stored in the D-latch. When next input spike occurs, the neuron then fires a spike, and the memristor is set to low resistance. The typical waveform of the memristive neuron is shown in Supplimentary Figure 2b. A suddenly change resistance of the memristor may result in a spiking. However, as the final recognition accuracy is determined by the statistic amount of spikes, maybe thousands of them, a random single spiking caused by the resistance change of memristor takes little effect on accuracy.

Supplimentary Figure 2a. Circuit schematic of a memristor neuron.

Supplimentary Figure 2b. Typical waveforms for the memristor neuron.

**Supplimentary Note 3: Details of Time Division Multiplexing Access (TDMA)**

In order to save the hardware resource, Time Division Multiplexing Access (TDMA) is used in the CNN. The proposed ANN processes receptive fields one by one to simplify the circuit design and control. When the neuron focuses on a certain receptive filed, the spikes which present the data in this receptive filed are generated. Synapses receive and modulate the frequency of spikes according to the weights. The memristive neuron receives spikes from all synpases and generates axon spikes which are monitored by MCU. Then the neuron focuses on next receptive field when MCU receives enough spikes or time out. The flow chart of TDMA is shown in Supplimentary Figure 3. In this work, the most important point is to verify the method for substantial reduction of amount of neurons by TDMA, i.e., one neuron can act as 784 physical neurons. For systematic simplicity, we used software-based neurons in other layers.

Supplimentary Figure 3. Flow chart of TDMA.

**Supplimentary Note 4: Response of the memristor neuron**

Supplimentary Figure 4 shows the measured number of spikes of each neuron in the first convolutional layer vs. the theoritically calculated number of spikes for each neuron in the CNN model for some ramdom selected channels in total 32 channels. Due to the randomness of memristor, the points are distributed on the both side of the 45 degree trend line , indicating the theoretical and expremental results are consistent. That also means that the CNN model in the memristive network based on the memristive neuron works correctly. Supplementary Fig. 4 shows the number of spikes outputted from the first convolution layer of the CNN, and these spikes are applied to the following first Maxpool layer, the second convolution and MaxPool layer, and finally to the full connection layer. Although some error rates may be introduced by memristance variations, the CNN shows excellent error tolerance and can successfully recognize the handwritten digits with an accuracy rate of 97.1%.

Supplimentary Figure 4. Actual response of neuron vs. the ideal spikes needed in the CNN model. The angle between the axial and the trend line is 45^°^.

**Supplimentary Note 5: Design of convolutional neural network based on memristor neuron**

The configuration of convolutional neural network used to recognize the handwritten digit is shown in Supplimentary Figure 5. The network contains two convolutional layers, two maxpool layers and two full connection layers. Each of convolutional layer is followed by a maxpool layer. The kernel size of two convolutional layers is 5×5; while the kernel size of maxpool is 2×2. The first convolutional layer has 32 convolution kernels, while the second convolutional layer has 64 convolution kernels. The first full connection layer has 3136 input neurons. The second layer has 1024 input neurons to realize better generalization capability. The weight parameters in convolutional layer and full connection layer are initialized by random number with truncated normal distribution(truncated value is 0.1). The bias parameters in convolutional layer and full connection layer are initialized by 0.1. The output generated by last layer follows One-Hot code, i.e., the largest number of spikes represents the recognized digit. A dropout layer (keep probability=0.5) is added between first full connection layer and second connection layer in order to improve generalization ability when the ANN is trained. The Stochastic Gradient Descent (SGD) is adopted to train the ANN, and the batch size is 100. For the throughput of the network, at 1k spikes/s, we have $6.478\times{10}^{-4}$ features/s for the first convolutional layer and $2.551\times{10}^{-3}$ features/s for the second convolutional layer; while at 1M spikes/s, we can have $6.378\times{10}^{-1}$ features/s for the first convolutional layer and $2.551$ features/s for the second convolutional layer.

Supplimentary Figure 5. Diagram of the Convolutional Neural Network for handwritten digits recognition.

**Supplimentary Note 6: Training the CNN based on memristor neuron**

The neural network is trained on the Platform of Tensorflow offline with Graphic Processing Unit (GPU). As memristor is a physical device, the weight of synapse in the network can only be selected as positive. The batch gradient descent is adapted to train the network. One handred handwritten-digit photographs were used to train the network for each batch. Supplementary Figure 6a shows the evolution of training error rate during batch training(a portion of training process, containing about 73 epochs for 40000 trainning steps) in a randomly selected epoch and we have 300 epochs for 165000 trainning steps in total training process. Supplimentary Figure 6b shows the error rate reduction with training epochs. After 165000 training steps of training process, the error rate declines to 1.3%, as shown in Supplementary Figure 6b.

Supplimentary Figure 6. (a) error rate vs. Batch number in a ranom selected epoch; and (b) Error rate vs. epochs.

**Supplimentary Note 7: Output of CNN layers when recongnizing digit “9”**

The output of first convolutional layer that contains 32 channels is shown in Supplimentary Figure 7a. Each channel is generated by a different kernel convoluted in the same original handwritten-digit photograph. The output of second convolutional layer that contains 64 channels is shown in Supplimentary Figure 7b. The second convolutional layer extracts abstracted infomation based on specific features in the first layer. As shonw in Supplementary Figure 8, Channels 1, 13 and 31 are blank, indicating that these channels did not extract valid information. The result is encoded by One-Hot code as shown in Supplimentary Figure 7c. The maximum amount of spikes exsits for digit “9” , indicating that the handwritten “9” is recongnized, which is consistent with the input.

Supplimentary Figure 7a. Result of the first convolutional layer of the CNN for recognizing digit “9”.

Supplimentary Figure 7b. Result of the second convolutional layer of the CNN for recognizing digit “9”.

Supplimentary Figure 7c. Result of the full connection layer for recognizing digit “9”. The amount of spike for “9” is the largest, indicating “9” is recongnized.

**Supplimentary Note 8: Recognition of handwritten digitals by the CNN**

Supplimentary Figure 8 illustrates the inference process of a handwritten-digit “9”. In this Figure, the gray scale represents the normalized value of neuron output, e.g. white represents “1” and black represents “0”. A photograph of handwritten “9” is input into the CNN. The first concovlutional layer extracts 32 features of original photograph and generates 32 channels whose dimension is 28×28 pixels. Then the maxpool layer compress the dimension of each channel to 14×14 pixels. The second convolutional layer extracts 64 channels of abstracted infomation, and then the maxpool layer compress the dimesion of each channel to 7×7. The hidden layer of MLP has 1024 neurons to map all the channels to an intermediate vector whose dimension is 1024. The output layer of MLP maps the intermediate vector to the output vector. The largest element in the output vector appears in the tenth position, indicating that the result of the recognition is “9”.

 Supplimentary Figure 8. Illustration of real time handwritten-digit recognition on the CNN based on the memristor neuron.

Supplimentary Reference

1. Hu, S. G. *et al.* Associative memory realized by a reconfigurable memristive Hopfield neural network. *Nature Communications* **6**, 7522 (2015).
